# Essential Role of Neuron-Enriched Diacylglycerol Kinase (DGK), DGKβ in Neurite Spine Formation, Contributing to Cognitive Function

**DOI:** 10.1371/journal.pone.0011602

**Published:** 2010-07-15

**Authors:** Yasuhito Shirai, Takeshi Kouzuki, Kenichi Kakefuda, Shigeki Moriguchi, Atsushi Oyagi, Kyoji Horie, Shin-ya Morita, Masamitsu Shimazawa, Kohji Fukunaga, Junji Takeda, Naoaki Saito, Hideaki Hara

**Affiliations:** 1 Laboratory of Molecular Pharmacology, Biosignal Research Center, Kobe University, Kobe, Japan; 2 Molecular Pharmacology, Department of Biofunctional Evaluation, Gifu Pharmaceutical University, Gifu, Japan; 3 Department of Pharmacology, Graduate School of Pharmaceutical Sciences, Tohoku University, Sendai, Japan; 4 Department of Social and Environmental Medicine, Graduate School of Medicine, Osaka University, Suita, Japan; 5 Laboratory of Pharmaceutical Technology, Kobe Pharmaceutical University, Kobe, Japan; Tokyo Medical and Dental University, Japan

## Abstract

**Background:**

Diacylglycerol (DG) kinase (DGK) phosphorylates DG to produce phosphatidic acid (PA). Of the 10 subtypes of mammalian DGKs, DGKβ is a membrane-localized subtype and abundantly expressed in the cerebral cortex, hippocampus, and caudate-putamen. However, its physiological roles in neurons and higher brain function have not been elucidated.

**Methodology/Principal Findings:**

We, therefore, developed DGKβ KO mice using the Sleeping Beauty transposon system, and found that its long-term potentiation in the hippocampal CA1 region was reduced, causing impairment of cognitive functions including spatial and long-term memories in Y-maze and Morris water-maze tests. The primary cultured hippocampal neurons from KO mice had less branches and spines compared to the wild type. This morphological impairment was rescued by overexpression of DGKβ. In addition, overexpression of DGKβ in SH-SY5Y cells or primary cultured mouse hippocampal neurons resulted in branch- and spine-formation, while a splice variant form of DGKβ, which has kinase activity but loses membrane localization, did not induce branches and spines. In the cells overexpressing DGKβ but not the splice variant form, DGK product, PA, was increased and the substrate, DG, was decreased on the plasma membrane. Importantly, lower spine density and abnormality of PA and DG contents in the CA1 region of the KO mice were confirmed.

**Conclusions/Significance:**

These results demonstrate that membrane-localized DGKβ regulates spine formation by regulation of lipids, contributing to the maintenance of neural networks in synaptic transmission of cognitive processes including memory.

## Introduction

Many growth factors, neurotransmitters, and other extracellular signals evoke a rapid, but transient rise in the amounts of diacylglycerol (DG) and inositol 1,4,5-trisphosphate (IP_3_) through the hydrolysis of phosphatidylinositol 4,5-bisphosphate (PIP_2_) by phospholipase C (PLC). DG regulates the functions of several enzymes including protein kinase C (PKC), Ras guanyl nucleotide-releasing protein (RasGRP), chimerins, and Unc-13. Then, the generated DG is phosphorylated by DG kinase (DGK) to produce phosphatidic acid (PA). PA is also important lipid second messenger regulating several enzymes including the mammalian target of rapamycin (mTOR) and atypical type of PKC. Therefore, DGK is thought to be a key enzyme regulating numerous cellular responses [1]–[4]. Indeed, recent researches using DGK KO mice clearly demonstrated DGK's importance in the immune system [5. 6], pathophysiological roles in the brain and heart [7], and insulin resistance in diabetes [8].

To date, 10 subtypes of DGK have been cloned and divided into 5 groups based on primary structural motifs. Type I DGKs (α, β, and γ) are characterized by calcium-sensing regions, the recoverin homology (RVH) domain, and EF-hand motifs, in addition to two cysteine-rich regions, C1A and C1B, homologous to PKC C1 domain. Among Type I DGKs, the β**-**subtype was cloned from a rat brain cDNA library in 1993 [9], showing its predominant localization in neurons, specifically in cerebral cortex, hippocampus, and caudate-putamen [9], [10]. This DGK subtype shows unique membrane localization [10], [11], and its expression in rat brain rapidly increases after 14 days of age [10], when synaptic maturation progresses. In addition, the control of splicing of the enzyme, which generates non-membrane bound variants differing at the C-terminus, is associated with mood disorders [11]. These results suggest that spatio-temporal regulation of DGKβ is important for neuronal functions and related to neuronal diseases, but its physiological role has not been elucidated.

Therefore, we investigated significance of DGKβ and its molecular mechanisms for neuronal functions using DGKβ KO mice which we developed, and show that spatio-temporal regulation of DGKβ results in proper spine formation contributing to long-term potentiation (LTP) and cognitive function including spatial and long-term memory.

## Results

**Figure 1:**
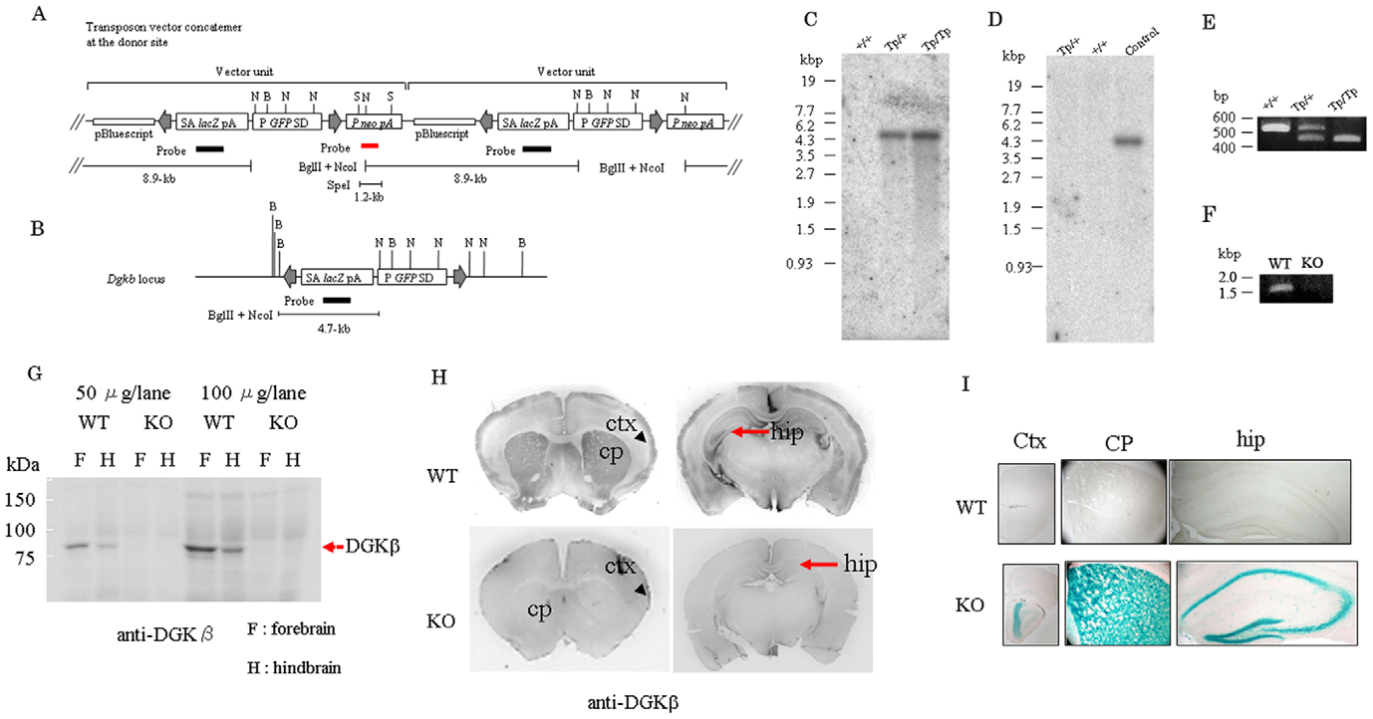
Characterization of DGKβ KO mice. (A, B) Vector DNA at the donor site and Dgkβ locus. SA, splice acceptor; pA, polyadenylation signal; P, cytomegalovirus enhancer/chicken beta-actin chimeric promoter; SD, splice donor; gray arrows, inverted repeats/direct repeats for transposase-specific binding; B, Bgl II; N, Nco I; S, Spe I. (C, D) Southern blot analysis of gene from WT (+/+), heterozygous (Tp/+), and KO mouse (Tp/Tp). Genomic DNA was digested with BglII and NcoI and detected with the lacZ probe shown in black. A single band in mutant mice indicates a single transposon insertion site segregated from the donor site (C). Similarly, genome DNA digested with Spe I was detected with the probe shown in red. Control represents genome from a control mouse cell line with one copy of neo. No band in the mutant mouse confirmed segregation of the donor site (D). (E) Typical result of PCR for genotyping. Bands at 455 bp and 540 bp are expected from the mutant and WT alleles, respectively. (F) RT-PCR. No band in RT-PCR indicates no mRNA of DGKβ in KO mice. (G) Western blotting using anti-DGKβ antibody. Proteins in the homogenate of hind- or fore-brain from WT and KO mice were separated by 7.5% SDS-PAGE, followed by transferring and immunostaining. (H) Immunohistochemistry using DGKβ antibody and frontal sections from WT and KO mice. (I) X-gal staining. The regions where DGKβ gene was mutated were determined by incubation with X-gal. Representative images showing cortex (ctx), caudate putamen (CP), and hippocampus (hip) of WT mice (upper) and DGKβKO mice (lower). Scale bar = 500 µm.

We produced DGKβ knock-out (KO) mice using the Sleeping Beauty transposon system [12]. This system utilizes the mobilization of a transposon cassette from the vector concatemer at the donor site and reinsertion of the cassette into other locations of the genome [13]. Southern blot analysis proved that KO mice did not possess a donor site and had only one insertion of the transposon cassette (Fig. 1A–D) and genotyping by PCR confirmed the KO mice (Fig. 1E). RT-PCR also indicates that there is no DGKβ mRNA (Fig. 1F). However, the insertion was between the 22^nd^ and 23^rd^ exons among 25 exons coding DGKβ, suggesting the possibility that a part of N-terminal region of DGKβ was still expressed. Therefore, we determined its expression at protein level with Western blot analysis and immunohistochemistry using the antibody specific for N-terminus of DGKβ [10]. Western blotting revealed that there was no expression of any part of DGKβ in the KO mice (Fig. 1G) and immunoreactivity was not detected in the hippocampus, cerebral cortex, and caudate putamen, where DGKβ was expressed in the wild type (WT) (Fig. 1H). Instead, β-galactosidase was expressed in these regions (Fig. 1I), indicating that the DGKβ gene was appropriately mutated by the splice acceptor-lacZ unit of the transposon vector.

**Figure 2:**
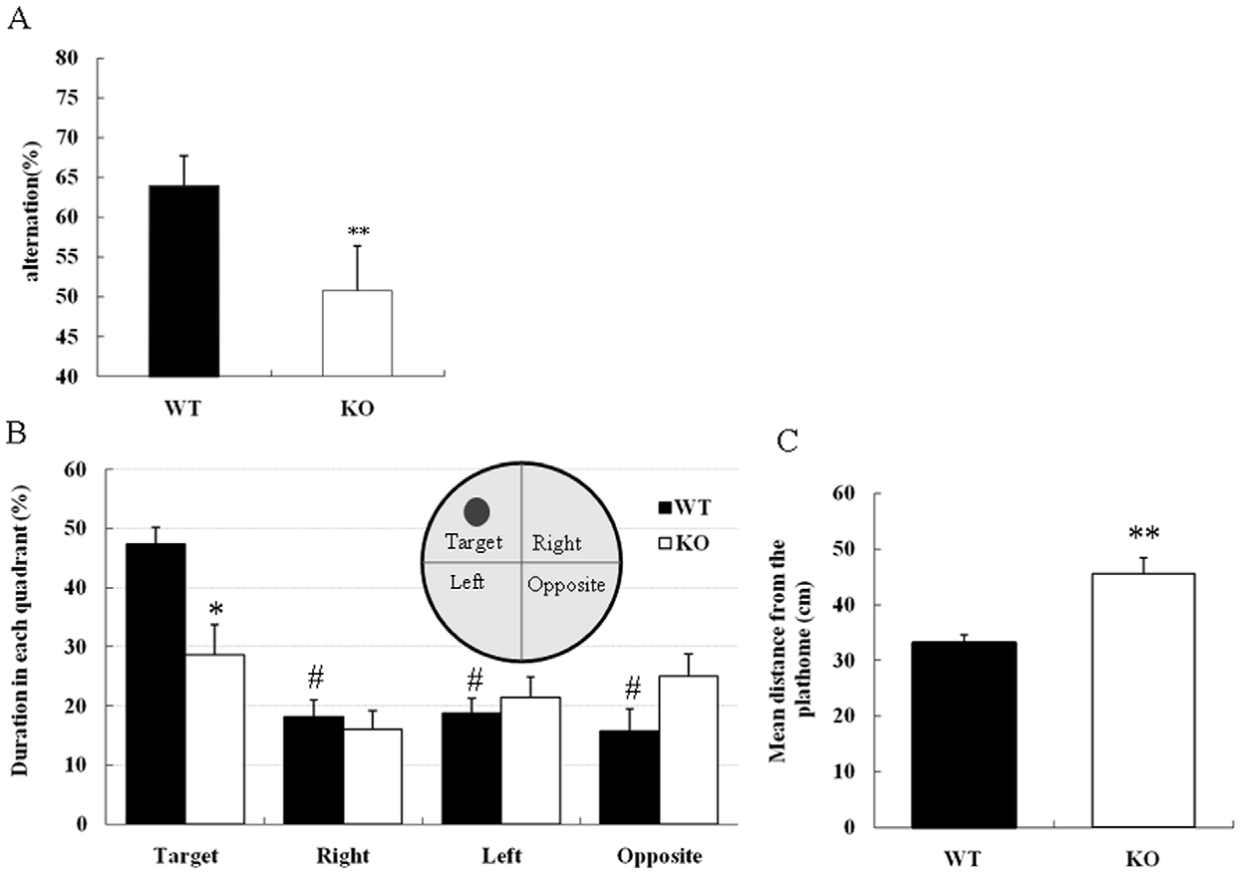
Impaired cognitive function in the DGKβ KO mice. (A) Spontaneous alteration behavior of Y-maze test. WT (n = 9) and DGKβ KO (n = 7) mice were placed at the end of one fixed arm and allowed to move freely through the maze during an 8-min session. ** represents P<0.01 vs. WT in the Student's *t*-test. (B and C) Morris water maze test of DGKβ KO mice (n = 9) and their WT litter mates (n = 8). Mice were placed in the opposite quadrant facing the wall and the time spent in each quadrant (B) and the mean distance from the platform (C) were measured. * indicates that the period DGKβ KO mice spent in the target quadrant was significantly different from that of WT (P<0.05, Student's t-test). # represents the probability that the difference in the time spent in each quadrant versus the target quadrant was significant (P<0.05 with one-way ANOVA and followed by Tukey's post hoc test.)

The KO mice were viable and fertile with no significant difference in weight. We subjected DGKβ KO mice to several learning and memory tests to determine the involvement of DGKβ in cognitive function, because mouse DGKβ was detected in the hippocampus by immunohistochemistry (Fig. 1), as reported earlier in Rat [10]. At first, we examined spatial working memory classified into short-term and hippocampus-dependent memory using a Y-maze test. In this test, DGKβ KO mice displayed a significant decrease in alternation compared to their WT litter mates (Fig. 2A). Next, we performed Morris water maze test to examine hippocampus-dependent reference memory. Here, we chose the appropriate method to present data because swim speed of DGKβ KO mice was higher than that of WT mice in Morris water maze test [WT; 16.6±1.1 sec (mean ± S.E.M, n = 8), KO; 19.6±0.5 sec (n = 9), p<0.05, student *t*-test]. The following data analysis is less affected by swimming ability such as swim speed and locomotor activity [14]. In a probe trial of this test, WT mice spent significantly more time in the target quadrant, indicating that they remember the place where the platform used to be (Fig. 2B). In contrast, DGKβ KO mice spent almost equal time in the four areas (Fig. 2B). Moreover, DGKβ KO mice swam further from the place where the platform used to be than WT mice (Fig. 2C). These results showed that DGKβ is necessary for cognitive function including spatial working memory and long-term reference memory.

**Figure 3:**
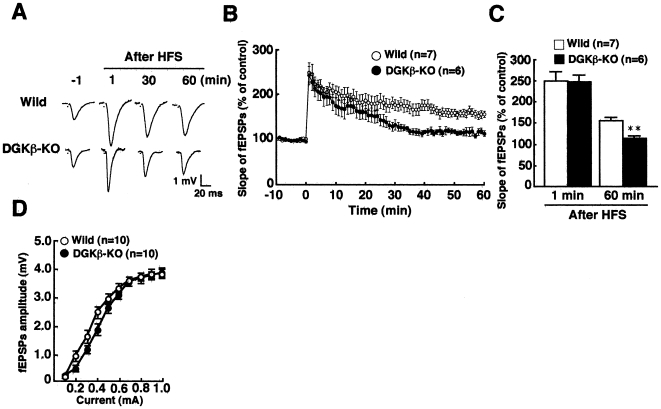
Impaired long-term potentiation (LTP) in the DGKβ KO mice. (A) Representative fEPSPs recorded from the CA1 region. (B) Changes in slopes of fEPSPs following HFS in the CA1 region from wild-type and DGKβ KO mice. (C) Level of LTP potentiation 60 min after HFS in the CA1 region from WT and DGKβ-KO mice. ** represents P<0.01 vs. the control of WT mice. (D) Input-output relationship between WT and KO mice. In the input-output relationship, amplitude of fEPSPs by stimulus intensity at 0.1 mA to 1.0 mA did not affect between DGKβ-KO mice and wild-type mice.

Next, we tested whether hippocampal LTP in the CA1 region is impaired in DGKβ KO mice since it is essential for hippocampus-dependent contextual and spatial reference memory. In control slices from WT mice, high-frequency stimulation (100 Hz, 2 trains) of the Schaffer collateral/commissural pathways induced LTP in the hippocampal CA1 region, which lasted over 60 min (156.6±7.1% of baseline at 60 min, n = 7) (Fig. 3A,B,C). As expected, a marked reduction of LTP was observed in DGKβ-KO mice (114.1±6.7% of baseline at 60 min, n = 6). (Fig. 3A, B, C). However, there was no significant difference in the input-output relationship between WT and KO mice (Fig. 3D), indicating that basal synaptic functions were not impaired. These results indicated that DGKβ is essential for hippocampal LTP in the CA1 region contributing to cognitive function and memory.

**Figure 4:**
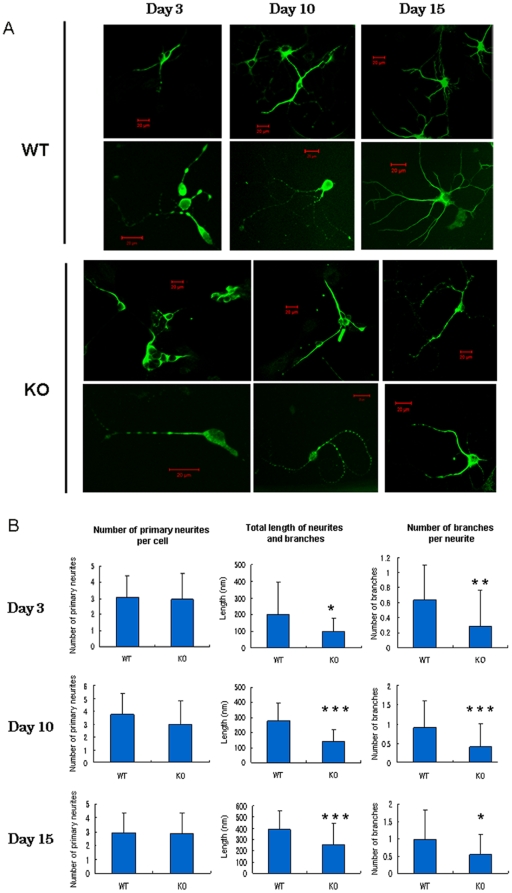
Impairment of neuronal branching in the primary cultured hippocampal neurons from DGKβ KO mice. Hippocampal neurons from WT and KO mice were cultured for indicated days. After fixing, the neurons were immunostained with MAP-2 followed by Alexa488-conjugated secondary antibody, and observed under confocal microscopy. (A) Typical images. Upper panels show lower magnification images and lower panels are magnified ones. Bars are 20 µm. (B) Static analysis. Following numbers of samples were analyzed: day 3, WT, n = 28 and KO, n = 27; day 10, WT, n = 37 and KO, n = 49; day 15, WT, n = 34 and KO, n = 31. *, **, and *** represent P<0.05, P<0.01, and P<0.005 vs. the control of WT, respectively.

**Figure 5:**
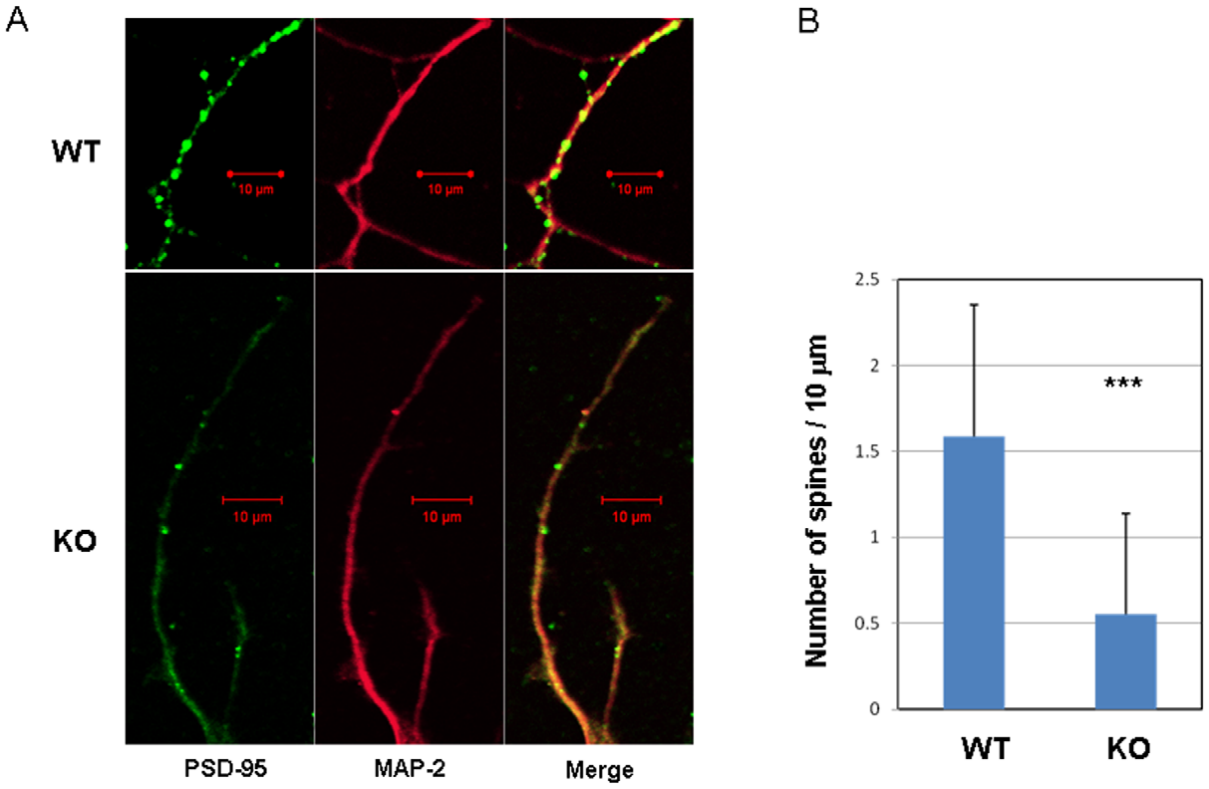
Impairment of spine formation in the primary cultured hippocampal neurons from DGKβ KO mice. Hippocampal neurons from WT and KO mice were cultured for 14 days. After fixing, the neurons were immunostained with PSD-95 (green) and MAP-2 (red) antibodies followed by Alexa488- and Alexa-594-conjugated secondary antibody. Following numbers of samples were analyzed: WT, n = 36 and KO, n = 50; *** represents P<0.005 vs. the control of WT, respectively.

**Figure 6:**
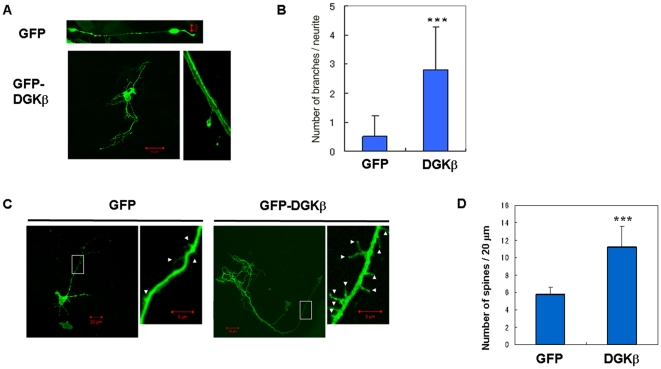
DGKβ-induced branching and spine formation in the primary cultured hippocampal neurons from WT mice. The mouse hippocampal neurons cultured for 3 days (A, B) or 10 days (C, D) were infected with respective viruses. After 48 h of infection, the cells were observed under confocal microscopy and analyzed using Neurolucida soft ware. (A) Typical images of primary cultured hippocampal neurons overexpressing GFP-DGKβ or GFP alone. (B) Statistical analysis of number of branches per a single neurite. n = 13 for GFP, n = 8 for DGKβ. (C) Typical images of spine-like structures in the primary cultured hippocampal neurons overexpressing GFP-DGKβ or GFP alone. Magnified images of squared area are shown in right panels. (D) Statistical analysis of spine-like structures. n = 18 for GFP and n = 15 for DGKβ. *** means P<0.005 vs. the control expressing GFP alone. Bars represent 5 µm (for magnified images) or 20 µm.

**Figure 7:**
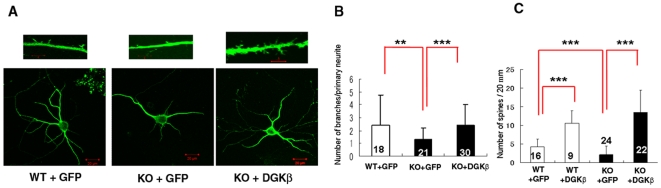
Rescue of impaired branching and spine formation in the primary cultured hippocampal neurons from KO mice by DGKβ overexpression. (A) Typical images of WT and KO primary cultured hippocampal neurons expressing GFP or GFP-DGKβ, and its distal dendrites with spines (upper panels). Bars are 20 µm or 5 µm (for upper images). (B, C) Comparison between numbers of branches per single neurite (B), and number of spines (C) in primary cultured hippocampal neurons from WT and KO mice. Numbers of analyzed samples are shown in the graph. **, and *** represent P<0.01 and P<0.005, respectively.

To explore the molecular mechanisms underlying impairment of memory and LTP, we compared morphological difference between the primary cultured hippocampal neurons from DGKβ KO mice and their litter mate WT mice. There was no significant difference in the number of primary neurites from a single cell body of the primary cultured hippocampal neuron from WT and KO mice (Fig. 4); those of WT and KO had about 3 at day 3 (corresponding to postnatal day 1), and the number did not change significantly until day 15 (corresponding to postnatal day 13). In contrast, the number of branches, as well as total length of neurites and branches, significantly decreased in KO mice in comparison with WT mice (Fig. 4). The decrease in the total length of neurites and branches was likely due to a lower number of branches in KO mice, indicating that DGKβ regulates branching of neurons. In addition, the number of spines was significantly reduced in KO mice (Fig. 5), demonstrating the importance of DGKβ in neurite spine formation. Reversely, we also checked the effect of DGKβ overexpression on morphological change of primary cultured hippocampal neurons. Overexpresion of GFP-DGKβ resulted in a significant increase in the number of branches and spines in the primary cultured neurons from WT mice (Fig. 6). Interestingly, unlike the neurons in the control group expressing GFP, the neurons overexpressing GFP-DGKβ possessed spines with very long neck (Fig. 6C, arrowheads in right image). Furthermore, overexpression of DGKβ in the primary cultured hippocampal neurons from KO mice improved branching; the number of branches was restored to the control level (Fig. 7A and B). Similarly, the number of spines was remarkably increased by overexpression of DGKβ to a level comparable to that of WT mice (Fig. 7B and C). These results demonstrated that DGKβ regulates neuron-specific morphological changes including neurite branching and spine formation.

**Figure 8:**
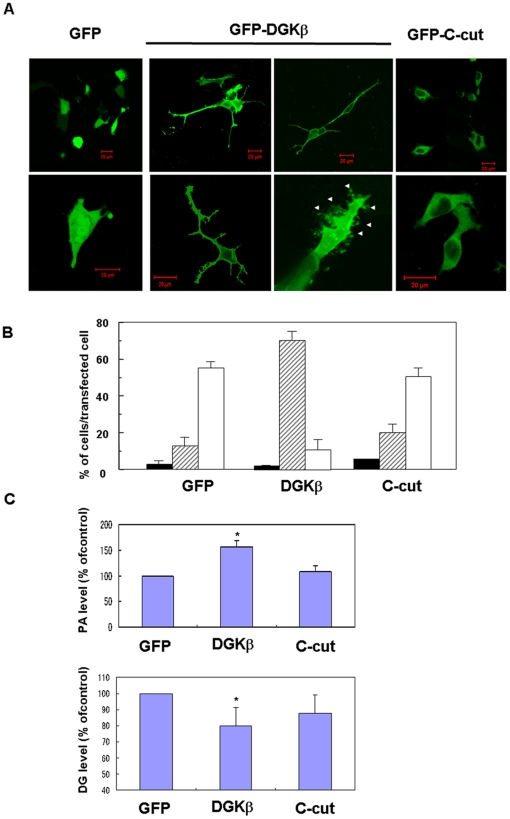
Disability of DGKβ splice variant form (C-cut) to induce branches and spines and its correlation to membrane PA and DG levels. (A) Typical images of SH-SY5Y cells overexpressing GFP-DGKβ, GFP-C-cut or GFP alone. Upper panels show lower magnification images and lower panels are magnified ones. Bars are 20 µm. Arrowheads indicate the position of spine-like structures. (B) Statistical analysis of morphological differences between SH-SY5Y cells overexpressing GFP-DGKβ, GFP-C-cut or GFP alone. More than 100 cells were observed in each experiment and three independent experiments were performed. The mean and SEM of number of the cells with one or two long neurites (closed column), several neurites with branches (slashed column), and no neurites (open column) are shown as percentage to the transfected cells. (C, D) Comparison of PA and DG level on the membrane of SH-SY5Y cells expressing GFP-DGKβ, GFP-C-cut or GFP alone. Membrane PA and DG were extracted from the cells and measured as described in the Materials and Methods. PA or DG contents are represented as mean of percentage to those of control cells expressing GFP alone. Each datum points represents mean with STD of three independent experiments. * means P<0.05 vs. the control expressing GFP alone (Student's T-test).

To further investigate molecular mechanisms of neuronal morphological change by DGKβ, we compared the effect of overexpression of GFP-tagged DGKβ and its splice variant type which lacks 32 amino acids from C-terminus (defined as C-cut mutant) on the morphology of neurons and lipids contents. In the neuroblastoma cell line, SH-SY5Y, DGKβwas mainly expressed on the plasma membrane (Fig. 8A), consistent with localization of GFP-DGKβ in primary cultured mouse hippocampal neurons (Fig. 6A magnified image) and endogenous DGKβ in primary cultured rat hippocampal neurons [10]. The cells overexpressing GFP-DGKβ induced more neurites with many branches than control cells expressing GFP alone (Fig. 8A and B) as seen in the primary cultured hippocampal neurons. In addition, the magnified image revealed that overexpression of DGKβ induced formation of spine-like structures (Fig. 8A, arrowheads in the magnified image). Statistical analysis showed that approximately 60% of the cells overexpressing control GFP had no neurites, while more than 70% of the cells expressing GFP-DGKβ had several neurites with branches (Fig. 8B), the proportion in the control being just 20%. Overexpression of wild type DGKβ significantly increased PA level on the membrane but decreased DG level (Fig. 8C and D) compared with control cells expressing GFP alone. In contrast, the C-cut mutant was localized in the cytoplasm and lost ability to induce neurites and spines (Fig. 8A and B). Coincidentally, overexpression of the C-cut mutant did not increase membrane PA and slightly decreased membrane DG level (Fig. 8C and D), although the mutant has enough kinase activity [11]. These results indicated that regulation of membrane PA and/or DG by membrane-localized DGKβ is a key of the neuronal morphological changes.

**Figure 9:**
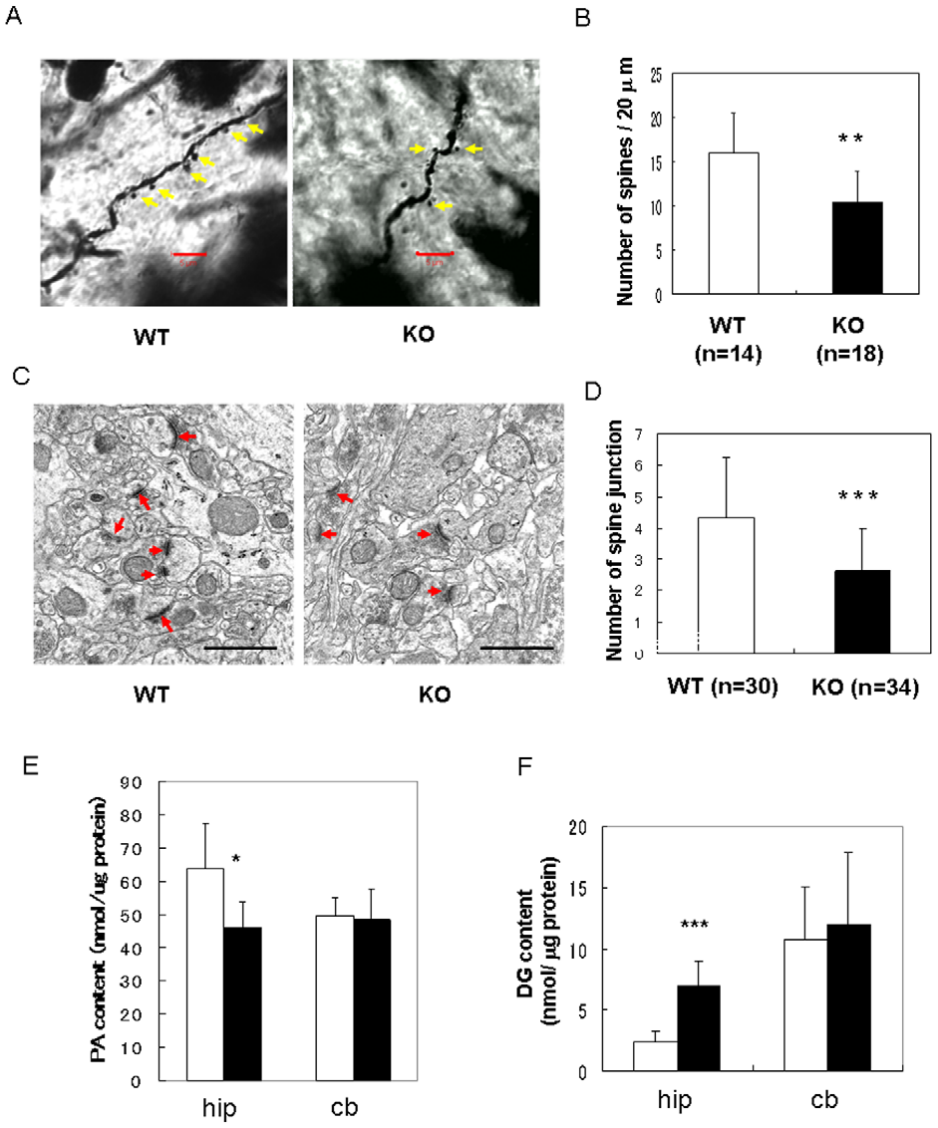
Abnormality of synapse density and PA/DG levels in the hippocampus of DGKβ KO mice. (A) Typical Golgi staining of hippocampal neurons at CA1 regions. Yellow arrows show spines. Scale bar represents 5 µm. (B) Comparison of synapse density in the CA1 hippocampal region. The number of synapses was counted and plotted. Each datum point represents the mean and SEM (WT, n = 14; KO, n = 18). ** represents P<0.01 vs. the control of WT. (C) Typical images of electron microscopy. (×243,000) Scale bar represents 5 µm. (D) Comparison of the number of synaptic junctions. Number of synaptic junctions with PSD in the micrographs was counted. Each datum point represents the mean and SEM (WT, n = 40; KO, n = 40). Red arrows indicate synaptic junctions. *** represents P<0.005 vs. the control of WT. (E, F) PA or DG level in the hippocampus and cerebellum from WT or KO mice. The hippocampus and cerebellum were dissected from WT or KO mice, and PA and DG contents were measured as described in the Materials and Methods. Each datum point represents mean with STD of three independent experiments. * and *** mean P<0.05 and P<0.005 vs. WT (Student's T-test).

These results suggested that phenotypes of the KO mice including the impairments of LTP and cognitive function were likely due to the disorder of neural networks caused by loss of DGKβ with imbalance of PA/DG contents. Therefore, we finally compared shape of neurons and lipids level in the CA1 region of the hippocampus from WT and KO mice. Golgi staining of hippocampal neurons indicated that spine density in KO mice was significantly lower than that in WT mice (Fig. 9A and B). The average number of synaptic junctions detected by electron microscopy was 4.3±2.0 per 14 µm^2^ in WT, while it was just 2.7±1.3 per 14 µm^2^ in KO mice (Fig. 9C and D). However, significant difference in the shape of dendrite and axons was not detected by immunohistochemistry and electron microscopic analysis. PA contents was reduced to about 70% in the hippocampus of the KO mice and DG contents increased 2.8.fold of the control, although there was no significant difference in the cerebellum where DGKβ is not expressed (Fig. 9E and F). These results implicated that loss of DGKβ disturbed PA/DG levels and synapse formation in vivo, resulting in impairment of LTP and cognitive function.

## Discussion

Here, we showed for the first time that DGKβ is essential for LTP in the hippocampal CA1 region and its-related cognitive function including spatial and long-term memory. The impairments are caused by, at least, dysfunction of spine formation in the KO mice based on the following results; number of spines decreased in the primary cultured neurons from KO mice (Fig. 5), overexpression of DGKβ remarkably increased spines (Figs. 6, 7 and 8), and spine density in the hippocampus of KO mice was lower than that of WT (Fig. 9). The importance of DGKβ in spine formation is supported by its postsynaptic localization reported by Hozumi et al [15], and by co-localization of DGKβ with PSD-95 in primary cultured rat hippocampal neurons (Fig. S1). In addition, DGKβ regulates neuronal branching because number of branches decreased in the primary cultured neurons from KO mice (Fig. 4), and overexpression of DGKβ induced branching (Fig. 6A and 7C). Indeed, number of branches from the control hippocampal neurons increased gradually at day 12 and 17 as shown in Fig. S2 (corresponding to postnatal day 10 and 15), coincident with expression level of endogenous DGKβ (Fig. S3). These findings suggest that the timing of DGKβ expression is well regulated to make precise neuronal network. However, physiological significance of DGKβ in the neuronal branching is still open to question because we did not find significant abnormality in the branching in the KO mice. In vivo, other DGK subtype(s) may compensate the branching. Possible candidate is DGKζ because it promotes neurite outgrowth via Rac 1 [16]. It is also noteworthy that there was no significant difference in the number of primary neurites of hippocampal neurons from WT and KO mice (Fig. 4) but DGKβ induces primary neurites only when overexpressed in the hippocampal neurons cultured just for 5 days (corresponding to P3) (Fig. S2), when endogenous DGKβ is not expressed yet (Fig. S3). These results indicate that DGKβ is not necessary for induction of primary dendrites and rather expression of DGKβ is suppressed in the early phase of neuronal development to prevent unnecessary neurite induction, confirming that temporal regulation of DGKβ function is critical for proper neuronal morphological change and development.

In addition to temporal regulation, spatial control of DGKβ is also important. Membrane localization of DGKβ with regulation of membrane PA/DG seems to be essential for its function of branching and spine formation, because the C-cut mutant, which has kinase activity but localizes in the cytoplasm, lost the ability to induce the morphological change and control PA/DG levels (Fig. 8). Interestingly, the C-cut mutant showed dominant inhibitory effect on the neurite induction and branching because overexpression of the C-cut mutant in the primary cultured neurons having neurites with branches, where endogenous DGKβ is already expressed, resulted in shrink of neurites and branching (Fig. S4). The dominant effect of the C-cut mutant on branching and spine formation may be related to symptoms of mood disorders and schizophrenia, because the C-cut mutant mimics the human splice variant form which is associated with these neuronal diseases [11]. Similarly, kinase activity on the membrane is necessary for the dendritic spine maintenance by DGKζ [17], [18]. Together with our results, production of PA and/or reduction of DG may be important for spine formation. Indeed, we confirmed that PA level was reduced and DG level was upregulated in the hippocampus of the KO mice (Fig. 9).

However, the precise molecular mechanisms underlying the spine formation by DGKβ is still unknown. DG signaling at the synaptic membrane may be a key for its function because DG regulates many enzymes including PKC and chimerin; PKC is considered to be a very important regulator of synaptic function [19], [20] and chimerin regulates dendritic spine density by biding to NMDA receptor [21]. On one hand, DGKβ may regulate neuronal morphogenesis through PA production because it is also a lipid messenger. Involvement of PA in spine formation and neurite induction is supported by the fact that overexpression of PLD also induces neurite outgrowth [22]. Possible candidates regulating spine formation at downstream of PA are mTOR and phosphatidylinositol 4 phosphate (PI4P)-5 kinase, because both enzymes bind to PA [23], [24] and regulate morphological changes of neurons; mTOR regulates dendritic morphogenesis [25], [26] and plasticity [27], and PI4P-5 kinase generates PIP_2_, which is one of the important regulators of spine formation [28]. In addition to PA and DG, the importance of lipid signaling for functional spine formation has been reported. For example, both PLC and PI3 kinase are involved in changes of the postsynaptic structure [26], [29], and lithium increase in synapse formation of the hippocampal neurons by depleting PI4P [30]. In any case, regulation of lipids at very restricted area seems to be important. Alternatively, DGKβ may be involved in the maintenance of structure of synapse based on the findings that overexpression induces spines with very long neck (Fig. 3D) and it accumulates on the perisynaptic site of neurons [15]. For the function to control structure of synapse, its interaction with actin may be a key because actin reorganization is generally involved in regulation of spine shape [31], [32]. Indeed, DGKβ colocalizes with actin filaments and its overexpression causes fragmentation of actin stress fibers [33]. Interestingly, there is a consensus sequence of actin binding, LKXXEX [34], in DGKβ, although direct binding between actin and DGKβ has not been reported. More experiments are needed to fully understand the mechanisms of the DGKβ-induced spine formation.

In conclusion, membrane-localized DGKβ regulates the morphology of neurons, specifically neurite spine formation, by regulation of lipids and its loss resulted in lower spine density causing impairment of LTP and memory related to cognitive functions.

## Materials and Methods

### Materials

SH-SY5Y cells and fetal bovine serum were purchased from RIKEN CELL BANK (Tokyo, Japan) and Invitrogen (San Diego, CA, USA), respectively. FuGene 6 transfection reagent was obtained from Roche Molecular Biochemicals (Indianapolis, IN, USA). Adenovirus encoding NSE-tTA and TetOp-GFP used were described previously [35]. The plasmids encoding Rat DGKβ were kindly given by Dr. Goto (Yamagata University, Yamagata, Japan). Rabbit anti-PSD-95 antibody was kindly gifted by Dr. Y. Fukata and M Fukata (National Institute of physiological Sciences, Okazaki, Japan). 1-stearoyl-2 arachidonoyl-2n-glycerol and L-α-PA monosodium salt from chicken egg were purchased from Biomol International (Plymouth Meeting, PA, USA) and Avanti polar Lipids (Alabaster, AL, USA).

### Animals

Pregnant mice (day 14) (C57BL/6JJmsSlc) for the primary culture were obtained from Japan SLC Inc. (Hamamatsu, Japan). DGKβ KO mice were produced using the Sleeping Beauty transposon system [12]. All mice were housed in a room with a 12-h light/dark cycle (light on at 08:00 a.m.) and had ad libitum access to food and water. Behavioral tests were performed between 10:00 a.m. and 6:00 p.m using 8–16 weeks old mice.

This study was approved by the Animal Experiment Committee of Gifu Pharmaceutical University (permission number; 08–114), the Institutional Animal Care and Use committee of Kobe University (permission number; 19-5-02) and the committee for Safe Handling of Living Modified organisms in Kobe University (H19-2). All procedures relating to animal care and treatment conformed to animal care guidelines of these committees.

#### Generation of DGKβ *KO* mice

Dgkβ-mutant mice were generated using the Sleeping Beauty transposon system as described previously^12^. Genotyping of the mutant mice was conducted by PCR using the following primers: 5′-GAACAGAACAACAATAGCTTATGTTC-3′ and 5′-CTTGTGTCATGCACAAAGTAGATGTCC-3′ for the mutant allele, and 5′-GAACAGAACAACAATAGCTTATGTTC-3′ and 5′-TAAGTGGATATTAGCCCAGAAACTTAG-3′ for the WT allele. 455-bp and 540-bp bands are expected from the mutant and WT alleles, respectively. PCR conditions were as follows: 25 µl PCR volume, 1 cycle at 95°C for 15 min; 35 cycles at 95°C for 1 min, 55°C for 1 min, 72°C for 1 min; and 1 cycle at 72°C for 7 min. DGKβ^−/−^ mice used for these studies were backcrossed to C57BL/6 mice for more than 9 generations.

#### Southern blot analysis

Genomic DNAs from mouse tails were digested with BglII and NcoI, fractionated on 1% agarose gel, and hybridized with the 1.4-kb lacZ probe, which is located inside the transposon vector. The lacZ probe was prepared by PCR-amplification from a plasmid DNA using following primers: 5′-AAATCCCGAATCTCTATCGTGCGGTGGTTG-3′ and 5′-GAAAGAAAGCCTGACTGGCGGTTAAATTGC-3′. To verify the absence of the vector concatemer at the donor site, genomic DNAs were digested with SpeI, fractionated on 0.8% agarose gel, and hybridized with the 0.8-kb neo probe, which is located in the vector backbone of the transposon vector. The neo probe was prepared by PCR-amplification from plasmid DNA using the following primers: 5′-TGGGATCGGCCATTGAACAAGATGGATTGC-3′ and 5′-CTCGTCAAGAAGGCGATAGAAGGCGATGCG-3′. Genomic DNA of a mouse cell line containing a single copy of the neo gene was used as a positive control to examine the sensitivity of the detection.

#### RT-PCR

Total cellular RNA was extracted from forebrain of WT and KO mice using commercial reagents (TRIzol Reagent; Invitrogen) according to manufacturer's instructions, and quantitated spectrophotometrically. Total RNA (0.2 µg) was reverse-transcribed into cDNA using the Super Script III First Strand Synthesis System for RT-PCR (Invitrogen) according to manufacturer's instructions. The cDNA was used as a template for following PCR to obtain quantitive value of DGKβ mRNA. Primers were synthesized commercially and the sequences were as follows. Forward'- primer was 5′-TGTGGACCCTTGAAGGACCATATTTTG and Reverse one wasCGGTTTCTTGTTCTTTTGATGAGGGAGCAG.-3′

#### Immunoblotting

Various ages of brain were dissected from developing mice and homogenized using a Polytron (Kinematica, Lucerne, Switzerland) in ice-cold homogenate buffer (250 mM sucrose, 2 mM EDTA, 20 mM Tris-HCl, 10 mM EGTA, 200 µg/ml leupeptin, and 1 mM phenylmethylsulfonyl fluoride, pH 7.4). A portion was taken for determination of protein concentration and the remainder was mixed with 3×SDS sample buffer. Equal amounts of brain samples were subjected to 7.5% sodium dodecyl sulfate polyacrylamide gel electrophoresis (SDS-PAGE) according to the method of Laemmli and the separated proteins were electorophoretically transferred to a polyvinylidine difluoride (PVDF) filter (Millipore, Billerica, MA, USA). The proteins separated by SDS-PAGE were transferred to a PVDF membrane and blocked with 5% skim milk in 0.01 M PBS containing 0.03% triton-X 100 (PBS-T). The membrane was immunostained with appropriate antibodies for 1 h at room temperature. After three rinses with PBS-T, the membrane was incubated with peroxidase-labeled anti-Rabbit IgG (Jacksons) or anti-mouse IgG (Jacksons) for 1 h at room temperature. After extensive washing with PBS-T, the immunoreactive bands were visualized using an enhanced chemiluminescence detection kit (ECL, Amersham Pharmacia Biotech).

#### Immunohistochemistry

DGKβ KO and WT mice were deeply anesthetized by the intraperitoneal injection of Nembutal (50 mg/kg). The mice were perfused with 0.9% NaCl through the left ventricle at a flow rate of 5–50 ml/min and then perfused with 250 ml of 0.1 M phosphate buffer (PB, pH 7.4) containing 4% PFA and 0.2% picric acid at 4°C. The brain was removed and immersed for 48 h in the same fixative. After washing with several changes of PB containing 30% sucrose for at least 2 days at 4°C, serial coronal sections of 20 µm in thickness were cut on a cryostat. These sections were immersed directly in PBS-T (0.1–0.3% Triton X-100) for at least 4 days at 4°C before use. The following steps were carried out at 25°C, unless otherwise specified. The frontal sections were washed for 5 min with PBS-T between each step. The frontal sections were preincubated with 0.3% H_2_O_2_ for 20 min to inactivate endogenous peroxidase activity, with 5% NGS for 20 min to block non-specific binding sites and then with 0.1% phenylhydrazine to inactivate endogenous peroxidase activity for 20 min. The sections were then incubated with the antisera against DGKβ (1∶2000) in PBS-T for 72 h at 4°C. After washing with PBS-T, the sections were incubated for an additional 2 h with biotinylated goat anti-mouse IgG (1∶1000) (Vector, Burlingame, CA), and then for 1.5 h with avidin-biotin-peroxidase complex (1∶1000) (Vector). After rinsing three times, the reaction product was visualized with 0.01% 3,3 diaminobenzidine (DAB, Sigma, CA, USA), and 1% nickel ammonium sulfate in 0.05 M Tris-HCl (pH 7.6) with 0.0003% H_2_O_2_. Finally, the stained sections were mounted on gelatin-coated glass slides, dehydrated by a graded series of ethanol and covered with Entellan (Merck, Whitehouse Station, NJ, USA) for observation, and then photographed under a light microscope (Carl Zeiss, Esslingen, Germany).

#### X-gal staining

Mice were anesthetized with Nembutal (50 mg/kg) and perfused with PBS (pH 7.4) until the outflow became clear, followed by 0.1 M PBS (pH 7.4) containing 4% PFA (Wako Pure Chemical Industries, Osaka, Japan) for 15 min. Brains were removed and postfixed in the same fixative for 24 h at 4°C. Brain sections were equilibrated in 25% sucrose and quickly frozen in Tissue-Tek O.C.T. (Sakura Finetek, Torrance, CA, USA, USA). After fixation with 0.2% glutaraldehyde and 1% formalin (Active motif, Carlsbad, CA), tissues were stained with 5-bromo-4-chloro-3-indoly β-galactoside(X-Gal) (Active motif) at 37°C for 24 h.

#### Construction of plasmid encoding GFP-DGKβ and its splice variant form (C-cut mutant)

The cDNA encoding DGKβ was produced by a polymerase chain reaction (PCR) with cDNA for DGKβ as the template. The sense and anti-sense primers used were 5′-ATAGGATCCATGACAAACCAGGAAAAATGG-3′ and 5′-ATAGGATCCTCATTCCTTGCTTCGGTTT-3′. For making the C-cut mutant, the same sense primer and anti-sense primer, 5′-TTGGATCCTATTGTGCATGGGGTCTG-3′, were used. The PCR products were first subcloned into pCR™ 2.1 (Invitrogen, San Diego, CA, USA) and sequenced. After digestion with Bam HI, the cDNA fragment was subcloned into the Bam HI sites of the pEGFP-C1 vector (Clontech Lab. Inc., Pal Alto, CA, USA) (designated as BS931).

#### Construction of adenovirus for GFP-DGKβ

The cDNA encoding GFP-DGKβ was cut out from BS931 by Nhe I/Mlu I digestion, followed by blunting using a blunting kit (Takara Corp., Tokyo, Japan). After adding a Hind III/Sma I adaptor, the cDNA fragment was subcloned into the Hind III site of a pShuttle vector (BSAd 103). After linearization by Pme I, the pShuttle vector with the cDNA encoding fusion protein of GFP and DGKβ was co-electroporated with the pAdEasy backbone vector into BJ5183 bacterial cells. The recombination was checked by Pac I cut and the plasmids were purified by CsCl-banding. Approximately 10 µg of the purified plasmids digested by Pac I were lipofected into 50–70% confluent HEK293 cells plated on a 6-cm dish using FuGene 6 (Roche). The cells were scraped off at 7 days post-transfection and re-suspended in 1 ml of PBS(−). After sonication, 50–70% confluent HEK293 cells in a T75 flask were infected using the supernatant and cultured in DMEM supplemented with penicillin (100 units/ml), streptomycin (100 µg/ml), and 10% horse serum (Gibco BRL). To amplify further, the infection of cells using 30–50% of the viral supernatant and 50–70% confluent HEK293 cells in T75 flasks was repeated. Finally, the adenovirus was purified by CsCl-banding and titrated.

#### Cell culture and transfection to SH-SY5Y cells

SH-SY5Y cells were cultured in DMEM/F-12 medium supplemented with 10% fetal bovine serum, penicillin (100 units/ml), and streptomycin (100 µg/ml) (Invitrogen Corp., Carlsbad, CA, USA). All cells were cultured at 37°C in a humidified atmosphere containing 5% CO_2_. The fetal bovine serum used was not heat inactivated.

SH-SY5Y cells (1×10^5^) were plated onto a glass-bottom culture dish (MatTek Corp., Ashland, MA) and then transfected with 2 µg of plasmid encoding GFP or GFP-DGKβ on the following day by lipofection using FuGene 6 according to the manufacturer's protocol. After culturing for 48 h, the cells were fixed with 4% paraformaldehyde (PFA) and 0.2% picric acid for 1 h at 4°C, and observed using confocal microscopy.

#### Primary culture of mouse hippocampal neurons

Fetuses were removed on embryonic days 17–18 from mice anesthetized by intraperitoneal injection of Nembutal (Abbott Laboratories, Abbott Park, IL, USA). Hippocampi were dissected and placed in Ca^2+^- and Mg^2+^-free HEPES-buffered Hanks salt solution (HHSS) at pH 7.45 (Invitrogen). Primary culturing of hippocampal neurons was carried out using Nerve Cell Culture System (SUMITOMO BAKELITE Co. Ltd., Tokyo, Japan). Briefly, hippocampal neurons were dissociated using dissociation solution and cultured using the glial-conditioned medium, in a glass-bottomed culture dish (Matek Corp, USA). Half of the medium was exchanged every 3 days with fresh medium.

#### Adenovirus infection to the primary cultured hippocampal neurons

After culturing for 3, 10, or 15 days, adenoviruses NSE-tTA, TetOp-GFP, or TetOP-GFP-DGKβ were applied to a dish culturing hippocampal neurons. After 1 h incubation, the medium was washed well and cultured for a further 48 h. After fixation with 4% PFA and 0.2% picric acid at 4°C and washing with PBS-T, the fluorescence of GFP was monitored under confocal microscopy.

#### Immunocytochemistry of the primary cultured hippocampal neurons

The hippocampal neurons dissected form DGKβ KO mice or their litter mates WT mice were cultured for 3, 10, or 15 days, and fixed with 4% PFA and 0.2% picric acid at 4°C. After washing with PBS containing 0.03% triton X-100 (PBS-T), the cells were tritonized with 0.3% triton X in PBS(−) for 20 min and then blocked with 5% normal goat serum (NGS) for 1 h. The neurons were incubated with anti-MAP-2 antibody (1∶2000) for 16 h at 4°C. After rinsing three times, the neurons were visualized with Alexa 488-labeled goat anti-mouse IgG (1∶1000), followed observation under confocal microscope.

#### Confocal microscopic analysis and image processing

The fluorescence of the GFP or Alxa 488 was observed with a confocal laser scanning fluorescent microscope (LSM 510 invert, Carl Zeiss, Jena, Germany) at 488-nm argon excitation using a 515–535-nm band pass barrier filter. The images were recorded as TIFF files. To count the number of neurites, branches, and spines, the image was analyzed with Neurolucida and Nurolucida Explorer software (MBF Bioscience, Tokyo, Japan).

#### Y-maze test

Spontaneous alternation behavior in a Y-maze was assessed as a spatial working memory task. The Y-maze apparatus consisted of three identical arms (length 40× width 10× height 12 cm). Each mouse was placed at the end of one fixed arm and allowed to move freely through the maze during an 8-min session. The sequence of arm entries was recorded manually. An alternation was defined as entering each of the three arms consecutively. The maximum number of alternations was thus the total number of arms entered minus two, and the percentage of alternations was calculated as (actual alternations/maximum alternations) ×100. The total number of arms entered during the session was also recorded.

#### Morris water maze test

A circular pool (diameter 120× height 45 cm) was filled to a depth of 30 cm with water (21–23°C). Four equally spaced points around the edge of the pool were designated as four starting positions. A hidden platform (diameter 10 cm) was set 0.5 cm below the surface of the water in a fixed position. Mice were placed in the water facing the wall and trained with 4 trials per day for 5 days. In each trial, the starting position was changed, and the mouse swam until it found the platform, or after 60 s was guided to the platform; the mouse was then placed on the platform for 15 s before being picked up. Twenty-four hours after the last training trial the mice were given a prove test without the platform. In this test, each mouse was placed in the pool once and allowed to search for 60 s. Mean distance from the original location of the platform and the time spent in the quadrant where the platform had been, was recorded using a video camera-based Ethovision XT system (Noldus, Wageningen, The Netherlands).

#### Electrophysiology

Preparation of hippocampal slices was performed as described previously [20]. Briefly, brains were rapidly removed from ether-anesthetized male WT or DGKβ KO mice (7–8 weeks old) and the hippocampi were dissected out. Transverse hippocampal slices (400 µm thick), prepared using a vibratome (microslicer DTK-1000), were incubated for 2 h in continuously oxygenized (95% O_2_, 5% CO_2_) artificial cerebrospinal fluid (ACSF) containing 126 mM NaCl, 5 mM KCl, 26 mM NaHCO_3_, 1.3 mM MgSO_4_ **•** 7H_2_O, 1.26 mM KH_2_PO_4_, 2.4 mM CaCl_2_ **•** 2H_2_O, and 1.8% glucose at room temperature (28°C). After a 2-h recovery period, a slice was transferred to an interface recording chamber and perfused at a flow rate of 2 ml/min with ACSF warmed to 34°C. Field excitatory postsynaptic potentials (fEPSPs) were evoked by a 0.05 Hz test stimulus through a bipolar stimulating electrode placed on the Schaffer collateral/commissural pathway and recorded from the stratum radiatum of CA1 using a glass electrode filled with 3 M NaCl. A single-electrode amplifier (CEZ-3100, Nihon Kohden, Tokyo, Japan) was used to record the responses, and the maximal value of the initial fEPSPs slope was collected and averaged every 1 min (3 traces) using an A/D converter (PowerLab 200, AD Instruments, Castle Hill, Australia) and a personal computer. After a stable baseline was obtained, high frequency stimulation (HFS) of 100 Hz with a 1-s duration was applied twice with a 10-s interval and test stimuli were continued for the indicated periods.

#### Golgi staining

The hippocampal region was cut out from the brains fixed as described above, and further immersed in 30% sucrose for 2–3 days. The tissue block was placed in 2% potassium dichromate for 2 days at 4°C and then in 2% silver nitrate solution for 2 days at 4°C in the dark. The block was cut into 60 µm thick sliced into distilled water. Finally, the sections were mounted onto slides, dried for 10 minutes, and dehydrated through 95% alcohol, 100% alcohol, clear in xylene.

#### Electromicroscopic analysis

The anesthetized DGKβ KO and WT mice were perfused with 0.9% NaCl through the left ventricle at a flow rate of 5–50 ml/min and then perfused with 250 ml of 0.1 M phosphate buffer (PB, pH 7.4) containing 2% PFA and 2% glutaraldehyde at 4°C. The dissected brain was sliced with into sections of 1 mm thickness and 4 mm square, and immersed in the same fixative. The slice was further fixed in the 2% osmium tetroxide in 0.1 M PB, dehydrated through a graded series of ethanol and embedded in Epon (quetol-812). The ultrathin sections were mounted on the mesh and stained with 2% uranyl acetate and Reynolds solution, then observed and photographed with a JEOL JEM1200EX electron microscope.

#### Measurement of PA and DG content

We measured membrane PA based on the methods by Aragones et al [36]. 5×10^5^ SH-SY5Y cells were lipofected with GFP-DGKβ, GFP-C-cut or GFP and cultured for 2 days. The cells were pre-incubated in phosphate-free medium for 30 min and then incubated with [^32^P]-monosodium phosphate (100 µCi/ml) for additional 2 h. Thereafter, the cells were harvested and lysed in 20 mM Tris-HCl (pH 7.5) containing 1 mM MgCl_2_, 1 mM EGTA, 1 mM PMSF, 20 µg/ml leupeptin. After sonication and centrifugation at 800×g, lipids including PA were extracted from the pellet, and separated by TLC. Spot of [^32^P]-PA was measured by BAS2500 (FUJIFILM, Tokyo Japan). To measure membrane DG, membrane lipids were extracted from the SH-SY5Y cells normally cultured. The amount of DG was determined by its conversion into [^32^P]-PA by Escherichia coli DGK in the presence of [γ-^32^P ]-ATP. DGK assay was performed described as previously [37].

To measure PA and DG in the brain from WT and KO mice, mice were anesthetized by the intraperitoneal injection of Nembutal and perfused with 0.1 M phosphate buffered saline. The brain was removed and a section (5 mm ×2 mm ×1 mm thick) was cut out from the hippocampus and cerebellum region of WT and KO mice. The sections were sonicated (30 s, input 5 by Tomi Seiko Co. Ltd's) in 100 µl of PBS (-). Protein concentration of the homogenate was measured by Bradford methods. DG and PA were extracted from the homogenates using chloroform and methanol as described previously [37]. A half of chloroform phase was dried up and subjected to measurement of DG using *Escherichia coli* DGK as described above. The half was subjected for measurement of PA using enzymatic reaction of lipase as described previously [38]. 1-steraroyl-2-arachidonoyl-sn-glycerol (1–100 pmol) and egg PA (1–40 pmol) were used for making standard carve, respectively.

## Supporting Information

Figure S1 Co-localization of endogenous DGKβ with PSD-95. The rat hippocampal primary neurons cultured for 21 days were fixed. Endogenous DGKβ was visualized with DGKβ antibody (1∶1000) followed by Alexa 594 conjugated anti-mouse IgG (1∶500), while PSD-95 was detected by rabbit anti PSD-95 antibody (Invitrogen) followed by Alxa 594 conjugated anti-mouse IgG (1∶500). Arrows indicate the spots where DGKβ colocalizes with PSD-95.
(9.42 MB TIF)

Figure S2 Temporally different effect of DGKβ overexpression on induction of primary neurite and neuronal branching in the primary cultured hippocampal neurons from WT mice. The mouse hippocampal neurons cultured for 3, 10, 15 days were infected with respective viruses. After 48 h of infection, the cells were observed under confocal microscopy and analyzed using Neurolucida software (plotted as day 5, 12 and 17, respectively). Number of primary neurites (A) and branches per single neurite (B) in primary cultured hippocampal neurons overexpressing GFP-DGKβ (closed square + solid line) or control GFP (open circle + dotted line) were compared. day 5; n = 13 for GFP, n = 8 for DGKβ, day 12; n = 18 for GFP, n = 15 for DGKβ, day 17; n = 8 for GFP, n = 12 for DGKβ. * and ** represent P<0.05 and P<0.01, respectively.
(9.04 MB TIF)

Figure S3 The ontogeny and localization of DGKβ in mouse brain assessed by immunoblot analysis (A) and immunohistochemistry (B). (A) Brain homogenates (50 µg protein) from mice of various ages were fractionated by SDS-PAGE, transferred to a PVDF membrane, and incubated with the specific antisera against the DGKβ and β-actin to show that equal amounts of protein were applied in each lane. The intensity of each sample was analyzed by NIH Image software and results are expressed as a percentage of the intensity at P21. P means postnatal. Each bar represents the mean of 3 separate samples. The typical immunoblot of DGKβ and β-actin is shown in the panels below the bar graph. (B) Immunohistochemistry showing DGKβ localization in frontal sections of mouse brain. ctx, cerebral cortex; hip, hippocampus; cp, caudate putamen.
(7.45 MB TIF)

Figure S4 Inhibitory effect of C-cut mutant on the neurite induction. GFP-C-cut or GFP was expressed in the mouse cortex primary neurons cultured for 8 days. Forty eight hours later, the cells were fixed and observed under confocal microscopy. Red arrow indicates that the neuron expressing GFP-C-cut did not have neurites, while black arrows show that the neurons expressing no GFP-C-cut have neurites (Upper images). Similarly, the neurons expressing GFP alone have neurites (lower images). Bars are 10 µm.
(7.07 MB TIF)
