# Supplementary figures and images for: Essential Role of Neuron-Enriched Diacylglycerol Kinase (DGK), DGKβ in Neurite Spine Formation, Contributing to Cognitive Function
Source: PLoS One. 2010 Jul 15;5(7):e11602. doi: 10.1371/journal.pone.0011602 (PMC2904696; doi:10.1371/journal.pone.0011602)

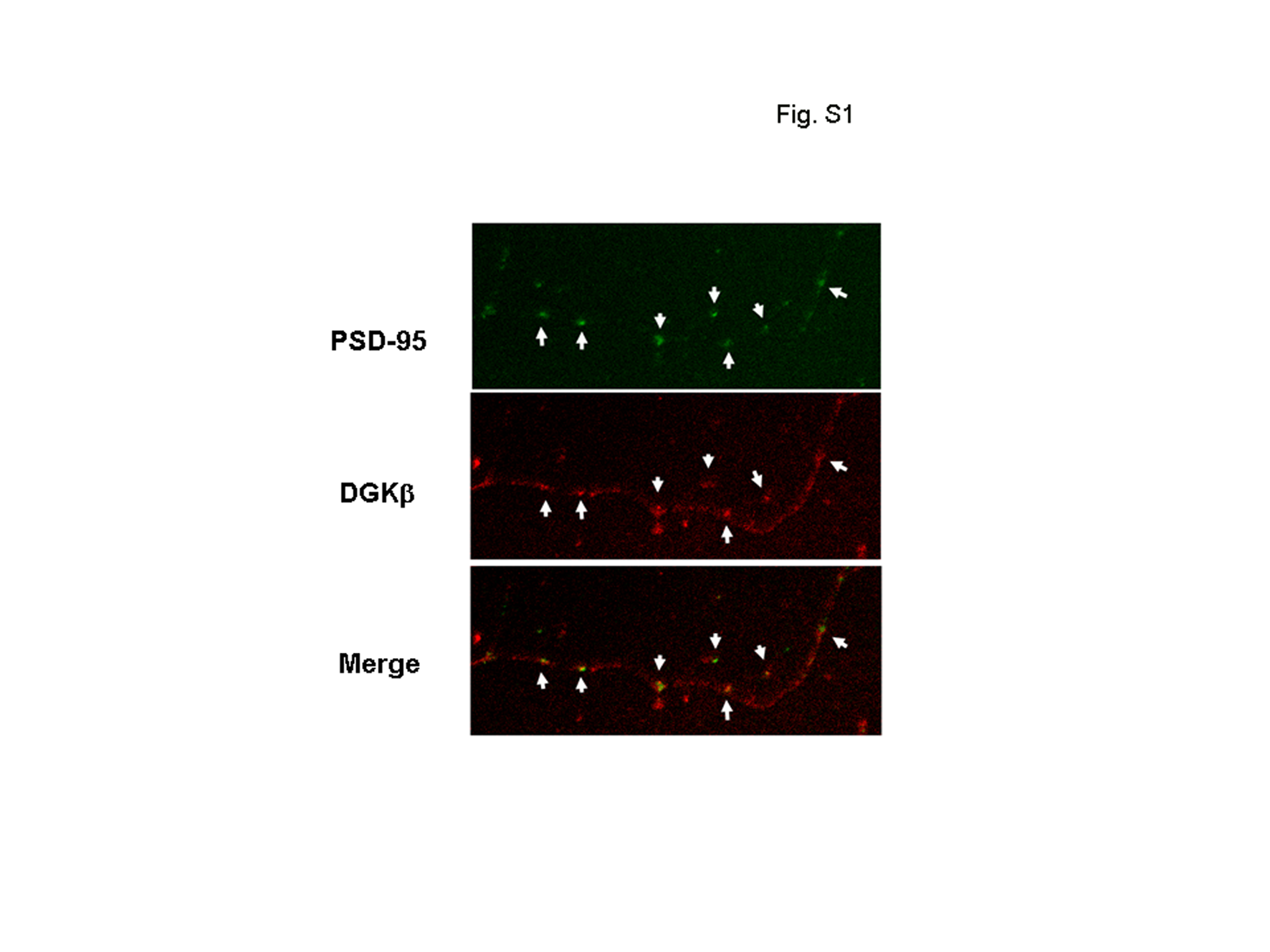

Supplement: Figure S1 — Co-localization of endogenous DGKβ with PSD-95. The rat hippocampal primary neurons cultured for 21 days were fixed. Endogenous DGKβ was visualized with DGKβ antibody (1∶1000) followed by Alexa 594 conjugated anti-mouse IgG (1∶500), while PSD-95 was detected by rabbit anti PSD-95 antibody (Invitrogen) followed by Alxa 594 conjugated anti-mouse IgG (1∶500). Arrows indicate the spots where DGKβ colocalizes with PSD-95. (9.42 MB TIF) [file pone.0011602.s001.tif]

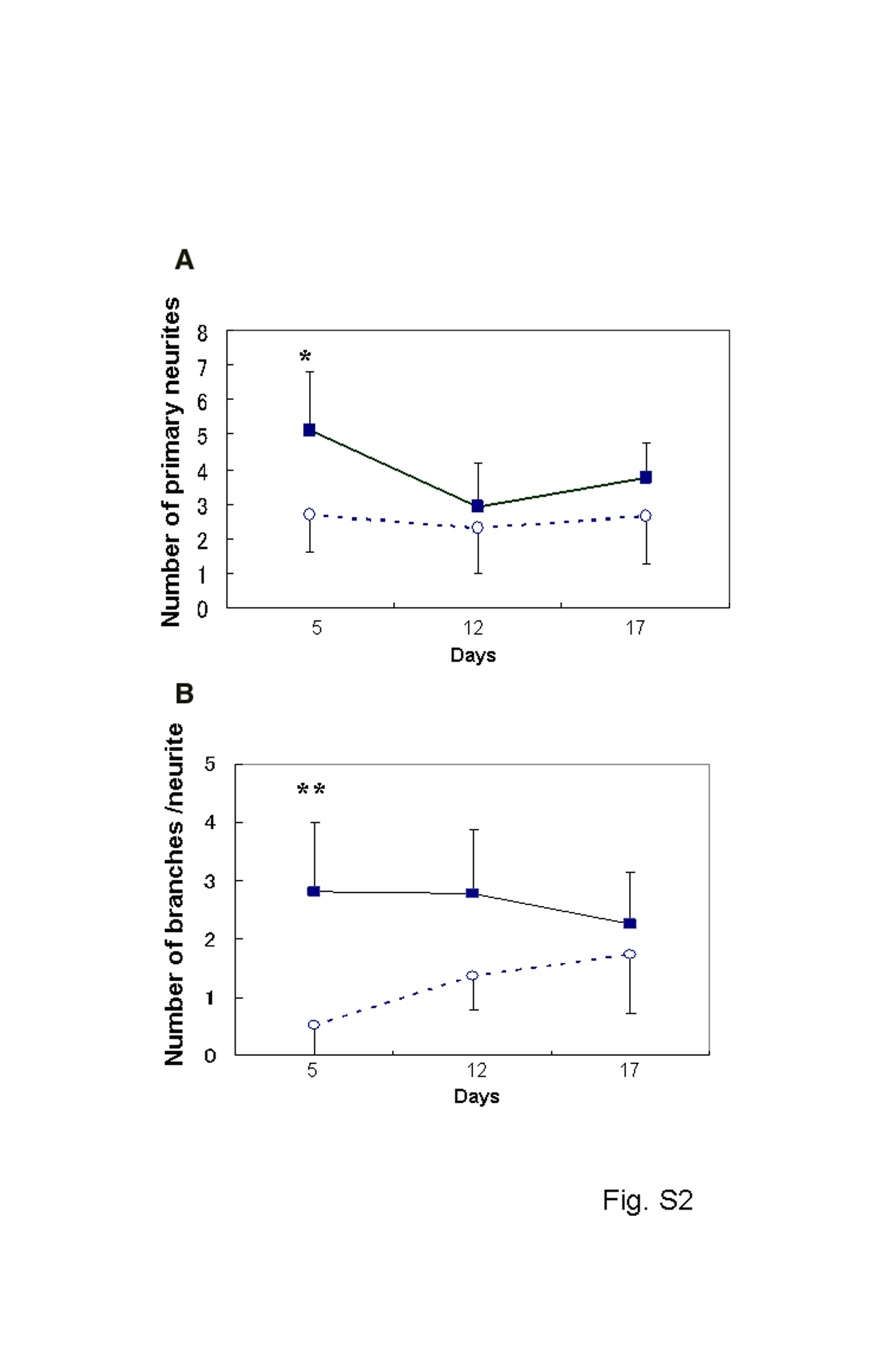

Supplement: Figure S2 — Temporally different effect of DGKβ overexpression on induction of primary neurite and neuronal branching in the primary cultured hippocampal neurons from WT mice. The mouse hippocampal neurons cultured for 3, 10, 15 days were infected with respective viruses. After 48 h of infection, the cells were observed under confocal microscopy and analyzed using Neurolucida software (plotted as day 5, 12 and 17, respectively). Number of primary neurites (A) and branches per single neurite (B) in primary cultured hippocampal neurons overexpressing GFP-DGKβ (closed square + solid line) or control GFP (open circle + dotted line) were compared. day 5; n = 13 for GFP, n = 8 for DGKβ, day 12; n = 18 for GFP, n = 15 for DGKβ, day 17; n = 8 for GFP, n = 12 for DGKβ. * and ** represent P<0.05 and P<0.01, respectively. (9.04 MB TIF) [file pone.0011602.s002.tif]

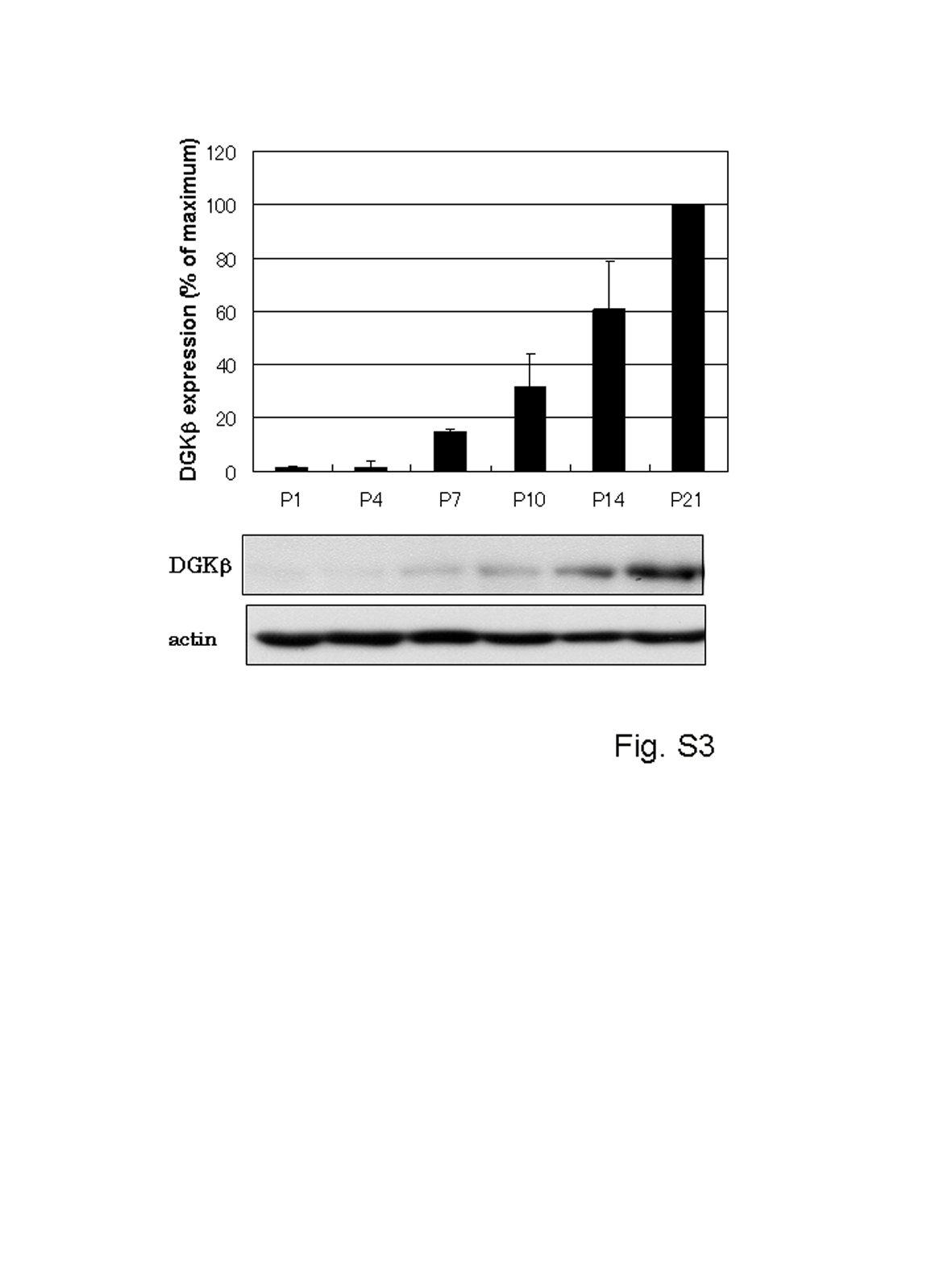

Supplement: Figure S3 — The ontogeny and localization of DGKβ in mouse brain assessed by immunoblot analysis (A) and immunohistochemistry (B). (A) Brain homogenates (50 µg protein) from mice of various ages were fractionated by SDS-PAGE, transferred to a PVDF membrane, and incubated with the specific antisera against the DGKβ and β-actin to show that equal amounts of protein were applied in each lane. The intensity of each sample was analyzed by NIH Image software and results are expressed as a percentage of the intensity at P21. P means postnatal. Each bar represents the mean of 3 separate samples. The typical immunoblot of DGKβ and β-actin is shown in the panels below the bar graph. (B) Immunohistochemistry showing DGKβ localization in frontal sections of mouse brain. ctx, cerebral cortex; hip, hippocampus; cp, caudate putamen. (7.45 MB TIF) [file pone.0011602.s003.tif]

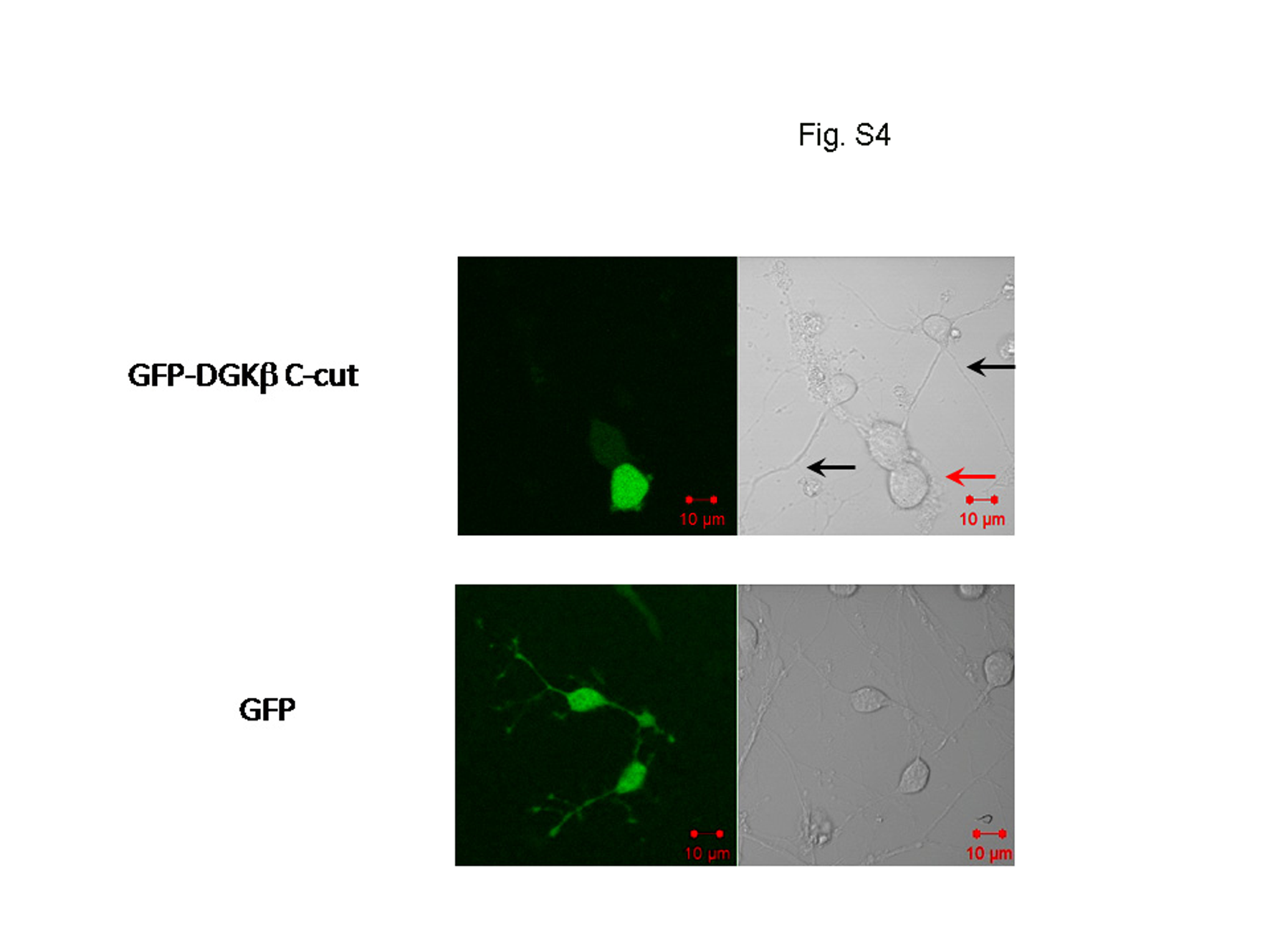

Supplement: Figure S4 — Inhibitory effect of C-cut mutant on the neurite induction. GFP-C-cut or GFP was expressed in the mouse cortex primary neurons cultured for 8 days. Forty eight hours later, the cells were fixed and observed under confocal microscopy. Red arrow indicates that the neuron expressing GFP-C-cut did not have neurites, while black arrows show that the neurons expressing no GFP-C-cut have neurites (Upper images). Similarly, the neurons expressing GFP alone have neurites (lower images). Bars are 10 µm. (7.07 MB TIF) [file pone.0011602.s004.tif]
